# Carbon Nanotubes (CNTs)-Reinforced Magnesium-Based Matrix Composites: A Comprehensive Review

**DOI:** 10.3390/ma13194421

**Published:** 2020-10-04

**Authors:** Somayeh Abazari, Ali Shamsipur, Hamid Reza Bakhsheshi-Rad, Ahmad Fauzi Ismail, Safian Sharif, Mahmood Razzaghi, Seeram Ramakrishna, Filippo Berto

**Affiliations:** 1 Department of Materials and Metallurgical Engineering, Amirkabir University of Technology, Tehran, Iran; Somayeh.abazari@gmail.com (S.A.); shamsipur@aut.ac.ir (A.S.); 2 Advanced Materials Research Center, Department of Materials Engineering, Najafabad Branch, Islamic Azad University, Najafabad, Iran; mahmood.razzaghi@gmail.com; 3 Advanced Membrane Technology Research Center (AMTEC), Universiti Teknologi Malaysia, Johor Bahru, Johor 81310, Malaysia; afauzi@utm.my; 4 Faculty of Engineering, Universiti Teknologi Malaysia, Johor Bahru, Johor 81310, Malaysia; safian@utm.my; 5 Nanoscience and Nanotechnology Initiative, National University of Singapore, 9 Engineering Drive 1, Singapore 1157, Singapore; 6 Department of Mechanical and Industrial Engineering, Norwegian University of Science and Technology, 7491 Trondheim, Norway

**Keywords:** magnesium, carbon nanotubes, composite, fabrication process, mechanical properties, corrosion behavior

## Abstract

In recent years considerable attention has been attracted to magnesium because of its light weight, high specific strength, and ease of recycling. Because of the growing demand for lightweight materials in aerospace, medical and automotive industries, magnesium-based metal matrix nanocomposites (MMNCs) reinforced with ceramic nanometer-sized particles, graphene nanoplatelets (GNPs) or carbon nanotubes (CNTs) were developed. CNTs have excellent material characteristics like low density, high tensile strength, high ratio of surface-to-volume, and high thermal conductivity that makes them attractive to use as reinforcements to fabricate high-performance, and high-strength metal-matrix composites (MMCs). Reinforcing magnesium (Mg) using small amounts of CNTs can improve the mechanical and physical properties in the fabricated lightweight and high-performance nanocomposite. Nevertheless, the incorporation of CNTs into a Mg-based matrix faces some challenges, and a uniform distribution is dependent on the parameters of the fabricating process. The characteristics of a CNTs reinforced composite are related to the uniform distribution, weight percent, and length of the CNTs, as well as the interfacial bonding and alignment between CNTs reinforcement and the Mg-based matrix. In this review article, the recent findings in the fabricating methods, characterization of the composite’s properties, and application of Mg-based composites reinforced with CNTs are studied. These include the strategies of fabricating CNT-reinforced Mg-based composites, mechanical responses, and corrosion behaviors. The present review aims to investigate and conclude the most relevant studies conducted in the field of Mg/CNTs composites. Strategies to conquer complicated challenges are suggested and potential fields of Mg/CNTs composites as upcoming structural material regarding functional requirements in aerospace, medical and automotive industries are particularly presented.

## 1. Introduction

**Figure 1:**
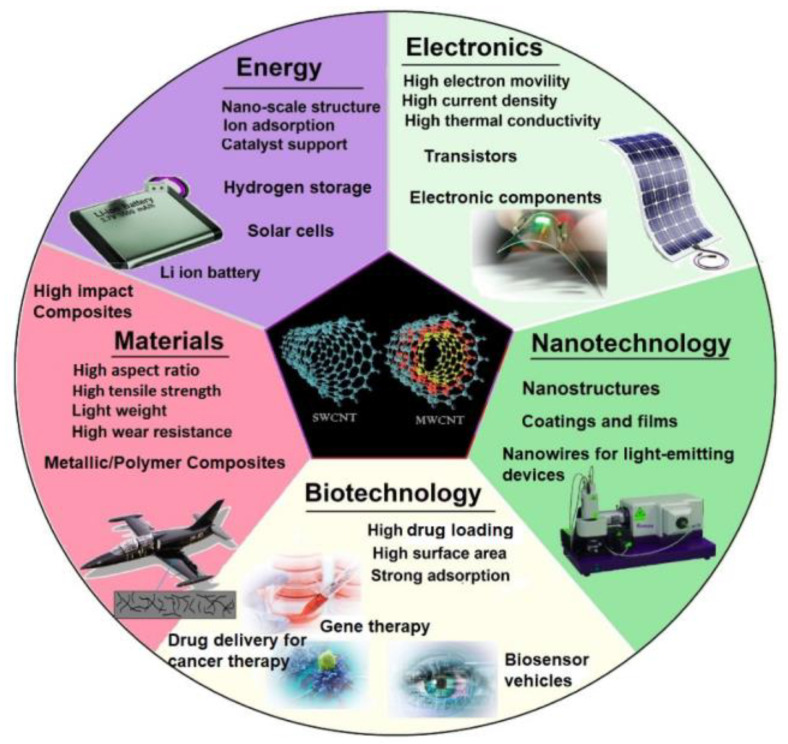
Schematic representation of properties and applications of carbon nanotubes (CNTs).

Modern technologies have a high demand for lightweight but robust materials in load-bearing applications. The density of magnesium (Mg) as the lightest structural metal is 1.738 g/cm^3^, which is about two-thirds of the aluminum density. The other specifications that make Mg attractive include its excellent specific mechanical properties, good ability for recycling, acceptable machinability, good damping response, nutritional characteristics, acceptable electromagnetic shielding, and abundance [1,2,3]. The aforementioned outstanding specifications of Mg and Mg alloys have made it a reliable candidate for use in aerospace, automotive, electronics, sports, and biomedical applications [4,5,6]. Nevertheless, Mg alloys have lower mechanical strength, especially at elevated temperatures, inferior creep behavior, and lower corrosion resistance compared to Al. Deformation of the materials with a HCP lattice, such as Mg alloys, at ambient temperature, is challenging because of their limited numbers of slip systems. Mg alloys can be strengthened by incorporating reinforcements like ceramic particles, carbon fibers, and metallic particles to fabricate Mg-based composites as a cost-effective manufacturing technique [7,8,9]. Recently, research has been focused on carbonaceous nanofiller materials, like graphene nanoplatelets (GNPs) and carbon nanotubes (CNTs) [10,11,12], for obtaining more attention regarding escalation of mechanical and electrical properties, as well as the required properties for biomedical applications. Among the carbon-based nanomaterials, CNTs have a wide range of applications on considering their large surface area, high aspect ratio, and unique mechanical, electrical, thermal, and optical properties [13,14]. CNTs are thermally and electrically conductive, robust like a diamond, while light, and have formability like graphite. Therefore CNTs have the properties of diamond and graphite [15]. It has been confirmed that CNTs exhibit ultra-high-modulus and strength, and also have anisotropic electrical conductivity when incorporated in a matrix, they can remarkably enhance fabricated nanocomposites. The CNTs are used in many other applications like sensors, transistors, field emission sources, voltage inverters, [16,17], solar cells, fuel cells, energy storage devices [18], drug-delivery systems and biosensors [19]. Therefore, new opportunities and research directions for developing novel metal matrix nanocomposites (MMNCs) could be opened up by applying nanotechnology in material science and engineering. The schematic representations of various beneficial properties along with applications of CNT materials are given in Figure 1.

Various studies have been conducted on CNT-reinforced MMNCs, particularly Al- and Cu-based matrix composites [20,21,22]. Although many polymer matrix nanocomposites reinforced by CNTs have been studied and great success has already been achieved on these nanocomposites [23,24,25], the challenge of incorporating nanocarbons materials in a metallic matrix and fabricating a sufficiently homogenous distributed structure still needs to be studied [23,26,27]. Another problem is the poor wettability of CNTs by melted metals because of a significant difference in surface tensions of metals and CNTs, which results in low interfacial bonding between CNTs reinforcement and the metal-matrix. In CNT-reinforced polymer-matrix nanocomposites, CNTs and polymeric matrices interact at a molecular level while in CNT-reinforced metal-matrix nanocomposites the molecular type of bonding is not possible. Great efforts have been made by researchers for achieving homogeneous dispersion of CNTs in the metallic matrix using different fabrication routes [28,29,30,31,32].

Hence, before introducing CNTs as an outstanding candidate for reinforcing the metals and alloys, the issues, including agglomeration of nanoparticles, and porosity in the synthesis and fabrication process, should be solved. Gravity-casting methods are at a risk of porosity, which could be a reason for inferior yield strength in nanocomposites. Nevertheless, by using the fabrication methods like squeeze casting, fabrication of the nanocomposites with low porosity is possible. The other is the agglomeration of the nanoparticles, which is an obstacle to achieving homogenous distribution. Concerning ultrasonic dispersion, de-agglomeration is dependent on how much power can be transmitted to the solution. Hence, the fabrication method is one of the key factors that should be considered in the development of MMNCs [33]. There are different routes for synthesizing the Mg/CNTs MMNCs. Generally, different fabrication routes can be classified into two categories; solid-state and liquid-state processes. Two common large-scale methods that are applied for fabricating particulate reinforced Mg-based composites are powder metallurgy (PM), which belongs to the solid-state category and casting that is the liquid-state. Based on the literature, an extensive number of studies have been dedicated to fabricating Mg-based composites applying PM and studying their mechanical characteristics [14,30,31,32,33,34,35,36]. The main steps in the PM methods, wherein all ingredients keep their initial solid-state (conditions), is blending the ingredients in powder form, compacting the blended powder, and sintering of the compacted specimen for achieving the part with minimum achievable porosity or maximum possible density. The predictable effect of decreasing the particle size in metal matrix composites (MMCs) to a nano-sized range solves some of the issues like poor formability, the low machining ability, and inferior fracture toughness of MMCs, and has enhanced their properties. Two main reasons for remarkably enhanced mechanical properties of MMCs are Orowan strengthening, and grain refinement mechanisms. High temperature creep-resistant properties of MMNCs are very attractive, especially when the matrix is a lightweight metal like Mg. The strengthening mechanisms which are involved in enhancing the mechanical properties of Mg-CNTs composites have been discussed carefully elsewhere [33,37]. The fast-growing nanotechnology in recent years has caused a noticeable decrease in the price of CNTs, especially for industrial grades [38]. Therefore, CNT nanofillers with reasonable prices are available as effective reinforcement for fabricating Mg-based nanocomposites with good mechanical properties. In this review article, the mechanical, corrosion and tribological properties of recent studies in the field of Mg/CNTs composites have been summarized. Moreover, the upcoming development and fabrication techniques of the Mg/CNTs composites have been offered from the point of view of the authors.

## 2. Carbon Nanotubes (CNTs)

**Figure 2:**
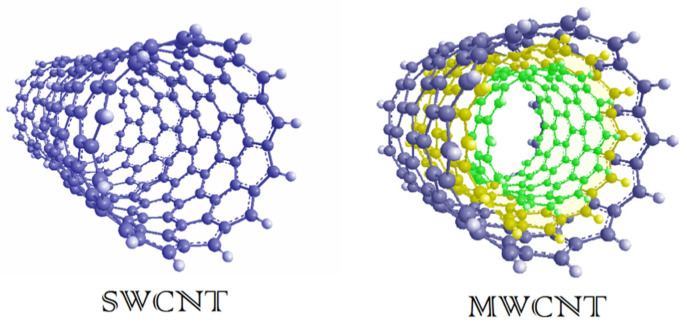
Schematic illustration of the morphological structure of CNT.

**Figure 3:**
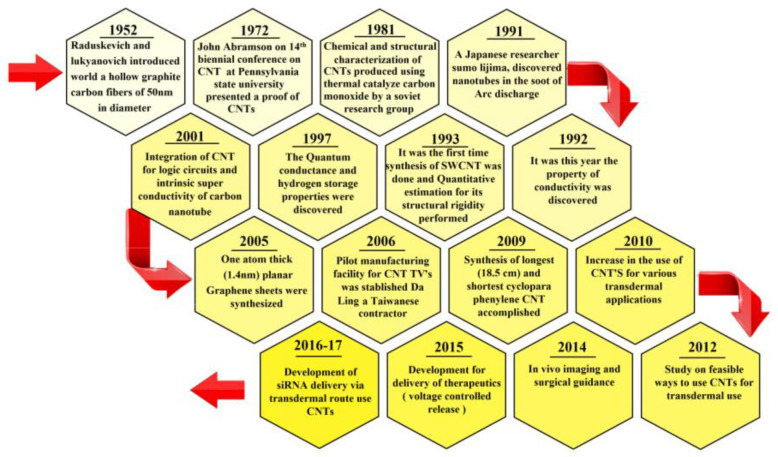
A brief historical timeline for CNTs showing the major developments that guided applications of CNTs [19].

In the early 1990s, Japanese physicist Sumio Iijima, accidentally discovered the presence of carbon molecules consisting of tubular coaxial nanotubes when he was discovering the spherical molecules of carbon in electric arc equipment [39]. The described layer of carbon, which is named graphene, is wound in cylinder shape for forming CNTs. Iijima discovered CNTs that were later called multi-walled CNTs (MWCNTs); it was named “Russian dolls” as they “nested” like them, which consisted of at least two graphitic layers having an inner diameter of about 4 nm [40]. Based on this report, Iijima and Ichihashi and their co-workers at IBM Almaden Research Centre in California synthesized single-walled CNTs (SWCNTs) [41]. The structures of MWCNTs and SWCNTs are illustrated in Figure 2. The SWCNTs were synthesized by using the same method as that Iijima et al. applied for fabricating the MWCNTs with diameters up to 100 nm, but with a slight modification. SWNTs are formed by the rolling of a single graphene sheet to form a cylindrical tube (diameters of between 0.4 and 2.5 nm) with excellent unique chemical, optical, electrical, and physical properties. SWCNTs have a better-defined wall with ultra-high surface area, which enables them to load multiple molecules by π-π stacking interactions [42]. Individual tubes had a diameter of as small as about 1 nm, and each was curled and looped rather than being straight [43,44]. As CNTs have many outstanding properties like very high strength, good flexibility, carbons with sp^2^ hybridization, and the ability to arrange themselves in a rope-like structure, they have grown to be one of the most extensively investigated nanostructured materials [45,46,47,48]. As shown in Figure 3, which presents a historical timeline for CNTs’ development, several milestones have been attained, and, remarkably, from the year 2010 onwards, a notable increase in the number of transdermal usages of CNTs started to arise [19].

CNTs as one type of one-dimensional nanomaterials have a large specific surface area in the range of 50 to 1315 m^2^/g. The CNTs’ radial dimensions are nano-sized, but the axial dimensions are micron-sized. The CNTs have an aspect ratio of 100 to 1000, which is far greater than that of traditional fiber materials, and have a density of as low as about 1.3 g/cm^3^. The C–C covalent bonds in the carbon rings are the most stable chemical bonds in nature with thermal conductivity of about 3000 W/m/K, which is comparable to the conductivity of the diamond. Besides CNTs’ electrical and thermal properties, superior mechanical properties, and their low density makes them highly capable of using them as a reinforcement to fabricate robust nanocomposites. Researchers have demonstrated that the CNTs have a Young modulus equal to that of the diamond and the strength of 100 times that of steel, and the density of as low as one-sixth of that of steel [49]. The reason for the outstanding mechanical characteristics of CNTs is the sp^2^ hybridized nature of C–C bonds [16,17]. Even for very low strength CNTs, the strength is as high as several GPa [15]. The Young’s modulus of CNTs is about 1 TPa, the highest one in all allotropes of carbon. Furthermore, due to having a hollow and closed topology, CNTs have a good bendability and plasticity [50]. Specifically, under external stress, CNTs could be shaped into a ring structure or bent to a small angle or then fully return to their original shape after removing the external stress. Even for the applied external stresses higher than the elastic deformation range, the deformed CNTs could tolerate more than 40% of tensile strain without a brittle fracture due to having Stone–Wales defects [51]. Every year large quantities of CNTs are produced through different techniques like chemical vapor deposition, arc evaporation, flame synthesis, laser ablation, electrolysis. Among the aforementioned methods, arc evaporation, laser ablation, and chemical vapor deposition are techniques are extensively used for the synthesis of CNTs [52]. The aforementioned outstanding characteristics of CNTs make them a fantastic material with numerous possible applications in different industries. In various industrial applications, the material applied for a specific purpose may have some poor properties that cause the degradation of the material and subsequently its breaking down. The exceptional characteristic of CNTs makes them capable of improving the essential characteristics of micro and macro materials and extending their durability through reinforcing them with CNTs.

## 3. Mg/CNT Nanocomposites

Recently, the demand in aerospace and automotive industries for decreasing the consumption of energy and emission of greenhouse gas has accelerated research into using the appropriate materials with low weight, and high strength [53]. Because it has an HCP crystal structure, Mg has poor ductility and strength at room temperature, compared with other metals with low density such as aluminum and titanium, which hinder its extensive use in industrial applications [54,55]. Because of the aforementioned issues, pure Mg could not be used in an industrial application, and the incorporation of alloying elements and reinforcing them with a secondary phase and developing cost-effective fabrication technologies seems necessary to solve the mentioned problems and enhance the Mg characteristics. The incorporation of low loading of nanoscale reinforcements could be a feasible option to fabricate the MMCs with high strength and other enhanced properties. Mg-based MMCs reinforced with particulate Al_2_O_3_, SiC, GNPs, and CNTs have been developed [28,56,57]. In particular, CNTs are more attractive than the other reinforcements because of their very high strength despite low density [5,6]. As CNTs have high strength (up to about 30 GPa) and high modulus, addition of them in the metallic matrix can result in increasing both the strength and stiffness of the fabricated MMCs [11,14,58]. Hence, CNTs have been proved to be one of the perfect reinforcements for MMCs. Lately, CNTs have attracted attention increasingly as a super reinforcement to improve the mechanical characteristics of monolithic Mg [11], Mg–6Al [59], and AZ31 and AZ81 [60,61] alloys. However, the mechanical characteristics of Mg/CNTs nanocomposites have been found to be much lower than what is predicted, such as MMCs reinforced graphene nanoplatelets (GNPs). This might be due to the low wettability of CNTs in a metallic matrix, which causes poor bonding between the CNTs and the Mg-based matrix. In addition, the issue could also be attributed to the special microstructure characteristic of the Mg composites because of CNTs’ incorporation.

Comparatively low mechanical properties, inferior corrosion resistance, and limited ductility are restricting factors for the application of Mg and its alloys. The corrosion of Mg is accelerated in contact with noble elements due to its low standard electrode potential of (−2.36 V) [60,61,62,63]. Therefore, the incorporation of impurities of Fe, Cu, or Ni, even in ppm level, can quickly increase the corrosion rate of the monolithic Mg [62]. Aung et al. [63] presented that CNTs might be incorporated into the Mg-based matrix for producing a composite with superior mechanical properties, but the influence of CNTs addition on the corrosion behavior was not completely known. Because of the distinct electrical properties of CNTs compared with other carbon allotropes, the effect of CNTs’ addition on the corrosion behavior of Mg is expected to be different. Fukuda et al. [64] studied the effect of CNTs’ incorporation on the corrosion behavior of AZ31 Mg alloy. To understand the corrosion behavior, immersion, and polarization tests, as well as the potential surface measurements as essential tools, were performed [65]. They showed that the galvanic corrosion between the Mg matrix and CNTs plays a big role in the corrosion properties of the fabricated composite. For optimizing the properties of the combination of Mg matrix and CNTs reinforcement, it is required to distribute the CNTs uniformly. Various methods have been used for optimizing the dispersion of CNTs in the Mg-based matrix.

## 4. Fabrication of the Mg–CNTs Metal Matrix Nanocomposites (MMNCs)

**Figure 4:**
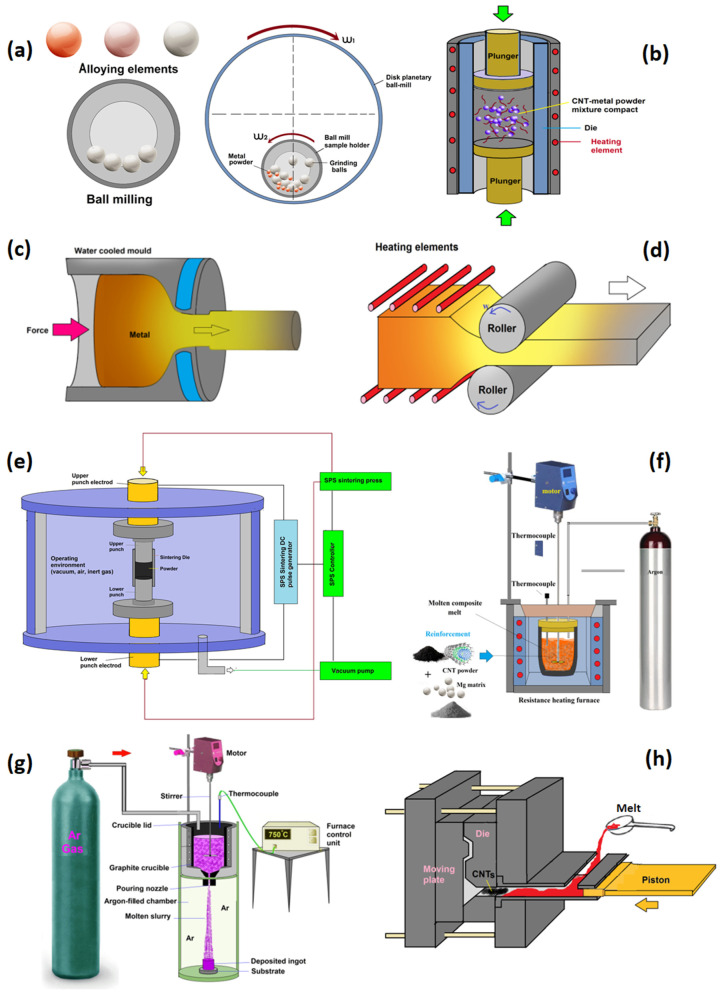
Different metallurgical methods for fabricating composites: (**a**) powder metallurgy [15], (**b**) hot press sintering, (**c**) hot-extrusion, (**d**) hot rolling, (**e**) spark-plasma sintering (SPS), (**f**) stir casting (SC), (**g**) disintegrated melt deposition (DMD) [11], and (**h**) high-pressure die casting [15].

CNT-reinforced Mg-based MMCs have been fabricated through different processing methods [31,35,36,53,55]. The PM process is the most popular and widely applied method for the fabrication of Mg/CNTs composites. For the metals with low melting temperature, like Mg and bulk metallic glasses, melting and solidification is an applicable process. Besides the aforementioned process, different efforts on indigenous processes have been made for fabricating Mg matrix-CNT composites. Figure 4 shows all of these processing techniques [11,15]. However, some critical issues still remain in fabricating CNT-reinforced MMCs: (1) attaining a homogeneous dispersion is difficult due to readily agglomeration of CNTs resulted by robust Van Der Waals forces, the attractive or repulsive interactions between the carbon atoms, (2) to maintain the structural integrity of CNTs during the manufacturing process, (3) controlling sound bonding and compact interfaces for attaining an appropriate load transfer between phases due to the poor wettability between carbon and metals, including Mg [60,66]. Different methods for distributing CNTs in metallic matrixes have been developed. Efforts for improving the distribution of CNTs have mainly focused on physical surface modification (like ball milling (BM) and high-energy ball milling (HEBM) processes), liquid dispersion routes (like ultrasonic treatment and mechanical stirring, Gemini dispersant), friction stir welding (FSW), and coating the CNTs surface with materials that can improve the wettability (like Ni [67], Si [68] and MgO [69]), CNTs doping (like boron-doped CNTs), and hybrid Al-CNT particles [70]. For the Mg/CNTs composites fabricated by the stir casting process, the dispersion of CNTs is rarely homogeneous in an Mg matrix because of the natural non-wettability of CNTs in molten Mg [14,34]. Moreover, some of the mentioned techniques can damage the integrity and structure of the CNTs. As an example, the ball milling process can result in an excellent dispersion but can easily damage the CNTs’ structure and reduce their strengthening influence in MMCs.

### 4.1. Powder Metallurgy (PM)

The majority of the research on Mg/CNTs composites has utilized the PM process for fabrication [30,57,71,72,73]. The steps of the basic PM process process include mixing CNTs and Mg powders by mechanical alloying or grinding, and then consolidating by compaction followed by sintering, and finally, the post-processes like cold isostatic pressing (CIP), hot isostatic pressing (HIP), or spark plasma sintering (SPS). In most of the studies, a post-processing deformation like rolling, equi-channel angular processing (ECAP), and extrusion of the compacted composites were applied. Regardless of the procedure stages, the key attention has been on achieving appropriate reinforcement, by obtaining a uniform distribution of CNTs in the Mg-based matrix and adequate bonding on the interface of the Mg and CNTs. Different parameters of the PM process are briefly discussed in the following subsections.

#### 4.1.1. Ball Milling (BM) and Sintering

Mechanical alloying has been utilized for preparing the composite powder particles. As described in the previous sections, to overcome the challenges in the process for the fabrication of Mg/CNTs composites, many studies have tried to adopt modified steps in their approaches. Zou et al. [72] have synthesized Mg/CNTs composites with different CNTs loadings and overall porosities by a powder metallurgical method. The procedure includes powder blending, compacting, and sintering. At the first step, the as-received powders are mixed for obtaining a uniform mixture utilizing a planetary ball mill. In the next step, the blended powder is compacted into a die for producing the green compacts using a compressive pressure. Finally, the compacted parts are sintered for the formation of the final products using a furnace. Li et al. [74] fabricated Mg/CNTs composite by this route but their specific process conditions. Habibi et al. [70] indicated the reasonable homogenous dispersion of the hybrid Al–CNT nanoparticles in the Mg matrix with Al incorporation of up to 1 wt.% by PM. The process of synthesis included blending hybrid Al–CNT nanoparticles with pure Mg powder using a ball milling process, and then compacting the blended powder at a pressure of 97 bar (the load of 50 tons). The compacted parts were sintered applying hybrid microwave-assisted 2-directional sintering method in a temperature near the melting point of Mg and finally the sintered parts were hot extruded. Authors showed reasonable homogenous dispersion of the hybrid Al–CNTs nanoparticles in the Mg matrix when the Al loading was up to 1 wt.%. For the Al content higher than 1 wt.%, reinforcement clustering occurred. Goh et al. [30] fabricated Mg–CNTs nanocomposites successfully applying the PM process. Although the bonding between Mg matrix and the CNTs is likely to be purely mechanical, good adherence was seen between Mg and the CNTs and no chemical reaction happened.

#### 4.1.2. Ball Milling (BM) and Hot-Press Sintering (HPS)

Some studies have utilized the hot-press sintering (HPS) process of the blended powder, instead of conventional sintering [71,73]. The Mg/CNTs composites synthesized through the HPS process have shown enhanced mechanical properties (including compressive strength, hardness, and bending strength) resulting from the homogenous distribution of CNTs in the Mg-based matrix. The improvement in mechanical properties was attributed to grain refinement resulting in inhibition of grain growth resulting from the interlocking of CNTs [71,75]. Endu et al. [76] prepared AZ91/MWCNTs composites by a process including powder blending, and then vacuum controlled HPS and extruding processes. The researchers showed that the filler in the Mg-based matrix could considerably enhance the corrosion resistance. First, fine-grained powder of AZ91D magnesium alloy was prepared to apply an argon atmosphere-controlled ball miller. Then the MWNTs reinforcement was mixed with AZ91D alloy powder in the ball miller. Subsequently, the AZ91-MWNTs blended powder was HPS processed, and finally, hot extruded, followed by solution treatment and aging. This combination of treatment causes a significant escalation in the corrosion resistance of the composite. In another study [71], the first Mg powder was refined using the HEBM process in an argon atmosphere-controlled planetary ball, while stearic acid was applied as a control agent. Then the refined Mg powder was blended with 1 vol.% of the CNTs using the same planetary mill. Finally, the powder mixture was sintered using an HPS system. In another research [75], Mg-based composite containing 2 wt.% of CNT was fabricated using the dry blending of Mg and CNT powders followed by HPS at 600 °C in a vacuum-controlled atmosphere, under a pressure of 50 MPa for 30 min. Finally, the compacted pieces were post-processed using HIP at 600 °C for 60 min in an argon-controlled atmosphere. The results showed that a homogenous distribution of CNTs in the metallic matrix had been obtained.

#### 4.1.3. Spark Plasma Sintering (SPS)

The SPS process, as a comparatively new technique compared to the conventional sintering, has been explored by some researchers for fabricating Mg/CNTs composites [57,64,77]. In the SPS process, pulsed direct current is passed through the powder and a die, producing rapid heating and, therefore, significantly enhancing the sintering rate [78]. The sintered part with high density and low porosity is achievable in this process via spark impact pressure, joule heating, and electrical field diffusion. Normally, this process is appropriate for consolidation of nanopowders, without allowing enough time for grain growth. The SPS process and its post-processing deformation seem capable of consolidation. Although a short SPS process efficiently decreases the possibility of CNT agglomeration, clusters that may be formed in the preparation steps, including blending and compaction, could be carried over in SPS. Post-processing deformation has been found to solve the clustering issue. Also, at the processing conditions of SPS, CNTs may be damaged or react with the Mg matrix. The mentioned problems have not been appropriately clarified [14].

### 4.2. Semi-Powder Metallurgy (SPM)

The ball mill process is a complicated process due to the requirement for optimization of different parameters for achieving appropriate results. Moreover, it generates heat that can easily burn the Mg powder in the process. Mg-based composites with a homogenous distribution of CNTs could not be made by the PM process. Moreover, CNTs can be damaged during the process of milling, and it inherently breaks the composite material. For solving these issues, Gemini dispersant and introduced functional groups are used. A solution-based PM method is adopted for the fabrication of GNPs/MWCNTs reinforced Mg-based composites that are called semi powder metallurgy. In the SPM technique, the materials are mixed using some liquid solvents instead of a ball mill process [28,79,80]. Theoretical calculations and related experiments have shown that ultrasonic cavitation could generate a high local pressure of about 50 MPa [81]. The generated pressure can result in the appropriate distribution of CNTs in the Mg-based matrix and subsequently enhance the mechanical characteristics of the composite.

#### 4.2.1. Gemini Dispersant

**Figure 5:**
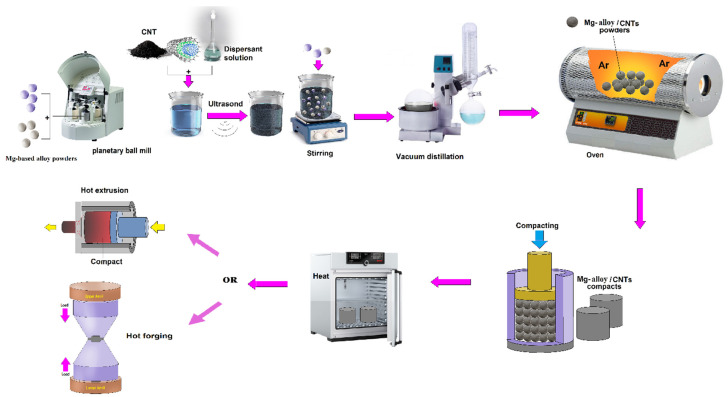
An illustration of fabricating Mg/CNTs using Gemini dispersant.

Among the main hurdles for the functional usage of CNTs is their agglomeration owing to the reliable van der Waals forces among CNTs, which has significant influence in distinctive characteristics of the individual nanotube and, likewise, deteriorates their functionality when applied in materials [60,66]. A crucial strategy to address this matter is the way in which to acquire uniform distribution of CNT in the matrix. One of the efficient techniques to distribute the CNTs in the Mg-based matrix can be the chemical modification of the CNTs, including covalent and non-covalent modification [82,83]. Although the covalent treatment could effectively distribute CNTs in the solution, this approach induces a covalent bonding surface which enhancing groups onto the surface of CNT via chemical or physical oxidation, which affect the CNTs’ structural integrity [82]. Recently, a kind of dispersant called Gemini has attracted great consideration due to its unique structure [84,85]. The molecules of Gemini dispersant usually contain two amphiphiles combined by a chemical bond, and amphiphile generally involves hydrophilic groups, hydrophobic chains, and aromatic rings. The peculiar structure of Gemini dispersant makes it capable of adsorbing on the surface of CNTs by π-π stacking interaction and hydrophobic interaction between dispersant molecules and CNTs simultaneously, leading to excellent distribution effect of CNTs [64,80,86]. Hou et al. [87] presented an ionic Gemini dispersant that could efficiently and homogeneously distribute the CNTs without damaging its structure, and the dispersant could be removed simply by applying an annealing process that could prevent the introducing of impurities. They mixed MWCNTs and ionic-Gemini surfactant, 4,4´-di (n-dodecyl) diphenylmethane disulfate salt (C_12_-DSDM) in deionized water for obtaining the MWCNTs’ dispersion. The authors presented that the DSDM-modified MWCNTs were negatively charged, giving rise to electrostatic repulsion between the MWCNTs in aqueous solution. A better MWCNTs distribution was seen with increasing MWCNTs surface potential, and the distribution with high MWCNTs surface potential presents high distribution stability with no clustering was seen for more than 5 months [87]. In the next step of the process, the powders of Mg and Al were gradually added to the prepared MWCNTs distribution, which was diluted using moderate ethanol, and stirred by impeller for 20 min. Then Mg-Al-MWCNTs mixture was dried applying the vacuum distillation process, and the obtained mixture of powder was annealed in an argon-controlled atmosphere for removing the dispersant. After annealing of the mixture, it was compacted, heat-treated, and hot-extruded. The fabricated MWCNT reinforced composite was found to be outstanding with no voids and debonding [53]. Figure 5 demonstrates the fabricating process of Mg/CNT composites using Gemini dispersant schematically.

#### 4.2.2. Formation of Functional Groups at the CNTs’ Surface

CNTs are remarkably prone to aggregation because of the large specific surface area, high length to diameter ratio, and high Van der Waals force between the CNTs, which could increase the porosity of the fabricated composites [88]. Formation of functional groups, like carboxyls (–COOH) carbonyls (>C=O), and hydroxyls (–OH) at the CNTs surface, can improve their surface characteristics. The CNTs were homogeneously dispersed after sonicating in anhydrous ethanol. The applied treatment also caused to remove the impurities of the surface [67,73]. Rashad et al. [79] incorporated Mg, Al, and Zn powders to ethanol for making a slurry with Mg-3Al-1Zn composition. They subsequently added nickel-coated CNTs in loadings up to 1.5 wt.% to the Mg-based slurry using a stirrer (1500 rpm, >2 h) to achieve a homogenous dispersion. After the vacuum filtration process for achieving a preform, the composite powder was poured in a stainless-steel die and compacted via hydraulic pressure. Fatih Aydin et al. [89] first prepared Mg/MWCNT powders applying the SPM process, including distillation and ultrasonication in ethanol solution and then a stir casting process. Mechanical stirring was utilized for mixing powder and molten metal under an atmosphere-controlled condition.

### 4.3. Hot Extrusion

Hot extrusion is a process in elevated temperatures in which the material is forced via a die. This process prevents work-hardening. Nevertheless, the manufacturing process involving hot extrusion needs a large amount of the powder for the fabrication of the composite in blending, milling, compacting, and lastly, extruding sub-processes [90]. The major disadvantage that has been seen in working with CNTs, at temperatures higher than about 500 °C, is the formation of carbides at the interface of metal and CNTs, which can decrease the strength of the composite. The mechanical properties of cast materials can also be enhanced using hot extrusion as a post-process. Shimizu et al. [91] fabricated high-density AZ91D/MWCNT composites applying BM, vacuum controlled HPS, and hot extrusion using different loadings of MWCNTs between 0.5 to 5 wt.%. No agglomerations of CNTs were seen in the fabricated composite for MWCNTs loading up to 1 wt.%. Nevertheless, the clustering happened for MWCNTs loading of 5 wt.%. The researchers showed that the short and linear CNTs impinged on the outer surface of Mg particles (about 100 μm) uniformly acted as an effective reinforcement for preventing deformation and consequently strengthening the composite. The extrusion method is an appropriate route for achieving superior material performance and improving the alignment of CNTs in the direction of extrusion. Harun Mindivana et al. [92] manufactured CNTs-reinforced Mg–6Al alloy using ball mill, cold press, and finally, hot extrusion processes with no sintering step. The CNTs loadings were 0.5, 1, 2, and 4 wt.%. Microstructural analysis of the fabricated parts showed that the CNTs on the Mg chips were present throughout the extrusion direction, and the homogenous dispersion of CNTs at the chip surface reduced with raising in the CNTs loading. Han et al. [57] showed that CNTs can promote the formation of twinning and weaken the basal texture in hot-extruded Mg-based nanocomposites.

### 4.4. Melting and Solidification Technique

As the earliest technique for fabricating MMC, the process including melting and solidification also has been applied for manufacturing CNTs reinforced composites. Numerous researches have been done employing the melting and solidification process to fabricate CNTs reinforced MMCs. This route can harm CNTs or cause the event of chemical reactions at the CNTs and metal matrix interfaces. Hence, this process is mainly preferred for fabricating the composites with a low melting temperature matrix. The other limitation for applying this route is that suspended CNTs are prone to form clusters because of forces of surface tension. Mg, as a metal with a comparatively low melting temperature has been appropriately processed through the melting/casting and solidification method [14,88,93].

#### 4.4.1. Stir Casting (SC)

Stir casting is the route mainly applied for the preparation of Mg/CNTs composites. Nevertheless, controlling this process could be difficult because of the high risk of Mg melt oxidation. Moreover, as mentioned before, CNTs have tended to agglomerate in the melt. The agglomeration of CNTs, segregation of secondary phases in the Mg matrix, and particulate fracture during mechanical agitation decrease the mechanical properties of the composite. The aforementioned problems could be prevented through controlling the parameters of the stir casting process for having appropriate mixing and dispersion of reinforcement in the Mg matrix, maintaining the vacuum in the crucible to prevent the Mg oxidation, and proper melt infiltration during the casting process [91,94]. Great efforts have been made by introducing better melt practices in order to attain improved distribution of CNTs in the Mg matrix. Gupta et al. systematically investigated the manufacture and assessment of mechanical properties of Mg-based composites reinforced with CNTs, metallic, or ceramic nanoparticles [93,94,95,96].

The microstructures of the cast parts have comparatively lower porosity as a significant characteristic in cast MMCs [97]. Kumar et al. [98] fabricated AZ91D-MWCNTs composite using the stir casting process. Based on the observations, they showed that the dispersion of CNTs was comparatively uniform without any defects and increasingly refined microstructural features. Moreover, AZ91D/MWCNTs (2 to 4 wt.% of loadings) composite was successfully fabricated, applying semi-automatic stir casting by Elvamani et al. [99]. MMCs display the dendritic cast structure containing CNTs particles in a eutectic matrix rather than the unreinforced Mg alloy with lower porosity. The grains are refined during the manufacturing process because of the continuous stirring and heating. MWCNT-reinforced AZ31 MMCs were prepared using the stir casting route by Abbas et al. [35]. They presented an increase in mass density with an increment of CNTs loading, which shows appropriate diffusion and compaction of the metal atoms. The porosity of the composites was reduced by increasing the weight percentage of CNTs, which could be due to the shearing of molten metal during stirring. The factors including the formation of effective linkages, good wettability in molten Mg, and uniform distribution of CNTs can result in decreased porosity.

#### 4.4.2. Disintegrated Melt Deposition (DMD) Route

The disintegrated melt deposition (DMD) process was adopted for dispersing nano-sized reinforcements in molten Mg-based matrix. The method includes mechanical stirring of reinforcement particles and Mg chips using an impeller in an argon-controlled atmosphere at a superheat temperature of 750 °C. The molten Mg is poured through a nozzle placed at the base of the crucible and disintegrated with argon gas jets. Subsequently, the fabricated ingot with the aforementioned method is extruded. Chan et al. [11] applied a novel process to fabricate Mg/CNTs composite utilizing an electromagnetic stirrer. In their route, an induction coil was located in the outer wall of the heating barrel for inducing a magnetic field to stir the molten metal. In this method, a mixed powder of Mg and CNTs was heated up to about 700 °C in a heating barrel, while stirring in an atmosphere-controlled condition. By vigorous stirring of liquid molten metal during solidification, it formed a slurry of fine solid floating on the metal melt. This slurry was then poured into a die for forming the composite. Paramsothy et al. [100] fabricated ZK60A/CNTs nanocomposite applying the DMD process, followed by hot extrusion. Homogeneous dispersion of CNT nanoparticles in the matrix was observed, which could be due to the dynamic deposition of composite slurry on the substrate followed by hot extrusion, argon gas disintegration of the metallic stream and minimal gravity-associated segregation because of the judicious selection of parameters for stirring.

### 4.5. Friction Stir Processing (FSP)

Friction stir processing (FSP) is a process, occurring in solid-state for obtaining a microstructure with fine-grains. This process is conducted employing a similar method as FSW, wherein a rotating-tool makes an extremely plastic deformed zone on a substrate. It is known that the friction zone contains fine and equiaxed grains formed because of dynamic recrystallization. Although the FSP process primarily has been used as a grain-refinement process, it is also an efficient method for manufacturing the composites. The utilized torsion or frictional force produced in the FSW process can cause the welding of the CNT and metal together for fabricating the CNT-reinforced metal matrix [32,101]. Based on the observation of microstructure, the main reasons for the acceptable AZ31/MWCNT composite surface hardness produced by the FSP process could be high interfacial bonding between the MWCNT reinforcement and AZ31 magnesium alloy matrix as well as the fine grain size [102]. Morisada et al. [32] utilized the FSP process for fabricating an Mg/CNTs composite. In this way, they made a groove in a bulk piece of Mg alloy, and put CNTs on it and then applied frictional force inside the groove with a rotating toll at 1500 rpm with different tool movement speeds. The researchers mentioned that CNTs are distributed in the metal matrix, and the grains are refined, but decreasing the grain size was not shown by the quantified results. Decreasing the tooling movement speed improved the dispersion of CNTs, which is attributed to increasing the time of mixing. Huang et al. [103] investigated the CNT-reinforced Mg-6Zn composites fabricated by stir-casting integrated with FSP. Their observations showed that the CNTs are uniformly dispersed in the matrix with acceptable bonding, resulting in grain refinement and enhancement in the mechanical properties of the matrix.

### 4.6. Spread Dispersion (SD)/Rolling Process (Micro-Nano Layered Structure)

**Figure 6:**
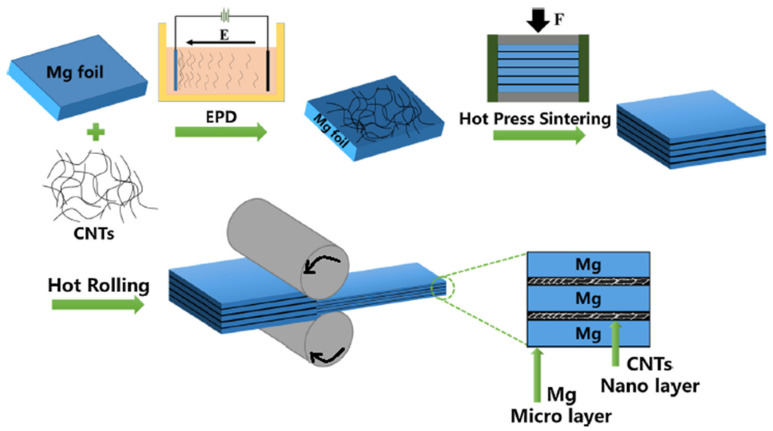
Schematic illustration of fabricating a micro-nano layered structure of Mg/CNTs composites [73].

The SD/rolling process is a newly developed fabrication method suitable for preparing Mg-based composites with a micro-nano layered structure. Xiang et al. [73] used this process for fabricating Mg/CNTs laminated composites. They combined for the first time electrophoretic deposition (EPD) of CNTs with 50 μm Mg foils for obtaining Mg-based composites with a micro-nano layered structure. Using the EPD process caused the uniform distribution of CNTs on Mg foils without any agglomeration. The thickness of the CNTs layer could be adjusted by regulating the deposition time. Furthermore, selecting the Mg foils with different thicknesses can control the parameters of the layered structure of the composites. Then, the stacked Mg foils were hot-rolled wherein a total thickness reduction of 60% was observed. Figure 6 illustrates the fabricating of a micro-nano layered structure of Mg/CNTs composites schematically [73]. The micro-nano layered structure makes huge back stress in the composites produced by CNTs layers that block the dislocations slip and thus attains higher strengthening and toughening efficiency compared to the conventional uniform composites. The enhancement in toughness is extracted through the escalation in energy needed concerning crack growth and significant Schmid factor regarding the basal slip system {0001} <11$\bar{2}$0>.

## 5. Strengthening Mechanisms and Mechanical Properties

Generally, the key aim of reinforcing metals with carbonaceous nanomaterials is the improvement in mechanical properties (including strength and Young’s modulus) of MMNCs. With the efficient transfer of stress through the interface of the metallic matrix and reinforcement, the achievement of strengthening and stiffening effects of carbon nano-fillers could be possible. The overall properties of Mg-based composites are influenced by the quality of the interfacial bonding between the CNTs reinforcement and the matrix, the formation of interfacial products, aspect ratio, and uniform distribution of CNTs in the matrix [104]. Moreover, the toughness is affected by crack deflection at the interface, and the ductility is influenced by the relaxation of peak stresses near the interface [105]. At the same time, the aforementioned characteristics are related to the utilized processing methods for fabricated MMNCs.

Better knowledge about the mechanisms of the strengthening in MMNCs can help in developing innovative composites [11]. Generally, four mechanisms have been suggested for explaining the strengthening effect of reinforcements in composites, as briefly described below.

**Load transfer:** as the most believed direct strengthening mechanism which can be happened through transferring the load from the matrix, to the reinforcement, through their interface, as proposed by Nardon and Prewo [106]. As normally the reinforcement is stronger and harder than the matrix, the mechanism can result in strengthening the composite. However, for achieving a considerable strengthening effect, load transfer needs a high aspect ratio of the nanomaterials [107,108,109]. Therefore, using reinforcement with higher strength results in a more efficient load transfer. The geometry of reinforcements, the bonding strength between the reinforcement and matrix, and volume fraction of incorporated reinforcements can influence the mechanism of load transfer. Compared with the conventional reinforcements, a very high aspect ratio of CNTs results in an efficient load transfer. The shear-lag model [110] has been extensively applied for estimating the mechanism of load transfer. In this model, the reinforcement fibers are assumed as 1D-axial load-bearing springs. In other words, in the shear-lag model, a three-dimensional fiber is considered to act as a one-dimensional object. The shear-lag model has been applied for determining the stresses in a broken fiber in different degrees of complexity [111].

**Orowan mechanism:** this mechanism happens during the plastic deformation, resulting in the movement of dislocations [112]. Because of the existence of the distributed CNTs in the matrix of Mg, residual dislocation loops were created adjacent to CNTs following a dislocation bends and bypass the incorporate agent based on the Orowan mechanism. These loops result in substantial work strengthening degree that is beneficial to reinforce the composite matrix [34,36,55,70,73,74,103]. Creating this mechanism needs a small inter-particle spacing between the reinforcement particles, and a rod-shaped reinforcement makes a more efficient strengthening than a spherical-shaped reinforcement [113]. The Orowan mechanism is essential for the composites, which have reinforcements, with smaller size and shorter inter-particle spacing [114].

**Thermal mismatch:** for the happening of this mechanism, a significant CTE difference between the reinforcement and the matrix for generating a higher density of dislocation around the reinforcements is needed. When the CTE values of the matrix and reinforcement are different, thermal changes applied to a composite can produce a thermal strain and internal stress. For accommodating this thermal mismatch effect, dislocations are generated around reinforcement particles to decrease the stored energy. The value of the thermal strain is related to the dislocations density, which is made in the composite, and the higher density of dislocations results in a more effective strengthening of the composites [115,116]. Multiple strengthening mechanisms [58] because of the addition of CNTs can be represented as the equation below.

σ_c_ = σ_m_ + Δσ_LT_ + Δσ_TM_ + Δσ_Orowan_ (1)

where σ_c_ and σ_m_ are the strengths of the composite and matrix, correspondingly whereas Δσ_LT_, Δσ_TM_, Δσ_Orowan_ are the enhanced strength because of load transfer, Orowan, and thermal mismatch mechanisms.

**Hall–Petch strengthening:** this strengthening mechanism happens based on the refinement of the grains, and is another mechanism for explaining the enhancement in compressive yield strength (CYS) of CNTs reinforced MMCs apart from the above three mechanisms. The Hall-Petch effect can effectively occur when the composite is applied to a process that can refine the average grain size [53,66,69,103,117], and different fabrication processes result in different Hall–Petch coefficients [118].

Further to the simple reinforcement theories based on load transfer such as ‘‘rule of mixture”, we expect size-dependent reinforcement mechanisms to happen. The extremely high aspect ratio of the CNTs provides smaller inter-particle spacing in the matrix at low loadings of reinforcement in comparison with the conventional reinforcements like SiC. Hence, CNTs reinforcements can obstruct the movement of dislocations in the composite. Plastic deformation can only progress if the dislocations circumvent the obstacle occurred by Orowan mechanism or shear the CNTs. As CNTs have a small diameter, shearing seems to be the possible mechanism. This means that the dislocations are held up at the CNTs, and the stress concentration at the head of the pile-up group of dislocations causes the CNTs to be yielded by fracture or deformation. For the reinforcements with a high aspect ratio, dislocations cannot easily climb to circumvent the obstacle, so not only enhancement of flow stress and toughness is expected, but also a remarkable increase in creep resistance of the material could be achieved [119].

The most common strengthening mechanisms of Mg/CNTs composites are “CNTs pull-out”, “crack deflection”, and “CNTs bridging”. During the fracture of the materials, a large number of CNTs are extracted from the Mg matrix. At the same time, the CNTs pull-out suppresses the stress of the crack tip, which can slow the propagation of the crack. Also, the CNTs pull-out can form some new interfaces and require an external force to work, and consequently consuming additional energy for propagating the crack. The bridging of CNTs generates compressive stress on the crack surface to act against the applied stress, making prevention for further growth of the crack and resulting in increasing the toughness of the composites. The crack deflection dramatically extends the path of crack propagation, consuming high energy before the composites failure. Xiang et al. [73] studied Mg/CNTs composite applying the micro-nano-layered structure fabricating process and represented the fracture morphology of the Mg/CNTs composites showing a significant change from smooth cleavage to a rough, jagged fracture morphology. The strain of unreinforced Mg was localized, and the morphology of the fractured surface was smooth for it. However, for the Mg/CNTs composite, because of the higher rate of the strain hardening, the composite deformed more uniformly, and also CNT layers delayed the propagation of the cracks. Hence, the CNTs’ layers efficiently improved the toughness of the composite.

Many studies have investigated the mechanical characteristics of Mg/CNTs composites. Table 1 shows the summarized information about the mechanical properties of Mg/CNTs nanocomposites. Ding et al. [71] studied a type of CNTs reinforced MMNCs fabricated by PM. They showed that the Mg powder could be refined by using a long-duration high-energy ball mill process before blending with CNTs. The aforementioned refinement resulted in a homogenous distribution of CNTs into the Mg matrix. The sintered Mg/CNTs nanocomposite possess a grain size of around 2–25 µm, which was determined via optical microscopy. This value is almost similar to the particle size of the as-milled specimens (2–18 µm), implying that no substantial grain growth occurred after the sintering process. The evaluation of mechanical properties showed that the nanocomposite displayed comparatively high strength and reasonable malleability of about 11%, and compression and yield strengths of 504 MPa and 454 MPa, respectively. Li et al. [34] adopted a two-step process for fabricating Mg-MWCNT composites. In the first step, a block copolymer was used as a distribution agent for pre-dispersing the MWCNTs on Mg alloy chips. In the next step, the chips with the well-dispersed MWCNTs on their surface were melted and, at the same time, vigorously stirred. Then the molten Mg alloy/MWCNT composites were poured into a cylindrical mold for rapidly solidifying. Both CYS and ultimate compressive strength (UCS) have been enhanced remarkably up to 36% by only incorporating 0.1 wt.% MWCNTs to the Mg-based matrix. The researchers claimed that the strengthening mechanisms were Orowan strengthening, load transfer, and thermal mismatch and showed that the CNTs bridge the propagating cracks efficiently, hence improving the formability of the Mg matrix. Yuan et al. [69] studied the mechanical properties of AZ91 Mg alloy based composites reinforced by CNTs and coated by MgO. They showed that the incorporation of CNTs improved the mechanical properties of the matrix and claimed that the enhancement is due to the grain refinement, Orowan mechanism, CTE mismatch, and load transfer mechanisms.

Zeng et al. [120] fabricated AZ31-MWCNTs composite using a process including manual stirring of reinforcement into the molten Mg alloy. The result exhibited that MWCNT powders possess a diameter of around 30 nm with a length of 1–10 μm. Their outcome also revealed that in an optimal option, tensile properties of AZ31 matrix could be improved by incorporating 1 wt.% MWCNTs. At the aforementioned optimal CNTs loading, all of the evaluated properties of the composite, including tensile strength, hardness, Young’s modulus, and elongation, were remarkably improved compared with the AZ31 matrix. For the loadings higher than 1 wt.%, the tensile properties declined because of the clustering of MWCNTs reinforcement. Moreover, MWCNTs incorporation remarkably refined the grain size of the Mg–alloy matrix.

Hou et al. [53] showed that the incorporation of MWCNTs in Mg-9Al based could remarkably reduce the size of Mg_17_Al_12_ phase, from micron to nano size in length. The results of the tensile test showed that the ultimate tensile strength (UTS) and elongation of Mg-9Al based composite reinforced with 0.4 wt.% of MWCNTs were 355 MPa and 15%, correspondingly, showing 18% and 150% improvement compared to the unreinforced Mg-based matrix. The significantly improved mechanical properties were mainly due to the homogenous dispersion of the nanosized Mg_17_Al_12_ phase in the composite and the outstanding bonding between the MWCNTs reinforcement and the matrix. Goh et al. [30] fabricated Mg-based composites reinforced MWCNTs with a diameter of 20 nm with the loadings between 0.06 to 0.3 wt.%. The fabrication process included mixing MWCNTs with Mg powder in a blender followed by sintering and hot extrusion. MWCNTs clustering was seen in the composite reinforced with 0.3 wt.% of MWCNTs. The yield strength and ductility of the composites were enhanced by raising the MWCNTs loading. Nevertheless, CNTs incorporation has a minor influence on improving the UTS of the composites. For improving the distribution of MWCNTs in the matrix, Shimizu et al. [91] employed ball mill, HPS, and extrusion processes for fabricating AZ91D-MWCNTs composites. The composite reinforced with 1 wt.% of MWCNTs showed the maximum yield strength (YS) and UTS. It was revealed that the CNTs homogeneously impinged on the outer surface of Mg particles acted as an efficient reinforcement for preventing the deformation and consequently strengthening the composite. Liu et al. [121] fabricated 1.5 wt.% MWCNTs with a diameter of about 20–40 nm and length of around 1–5 μm reinforced Mg-based nanocomposite employing both processes of mechanical stirring and high-intensity ultrasonics. The researchers showed that the incorporation of 1.5 wt.% MWCNTs into Mg enhances its UTS, YS, and elongation.

**Figure 7:**
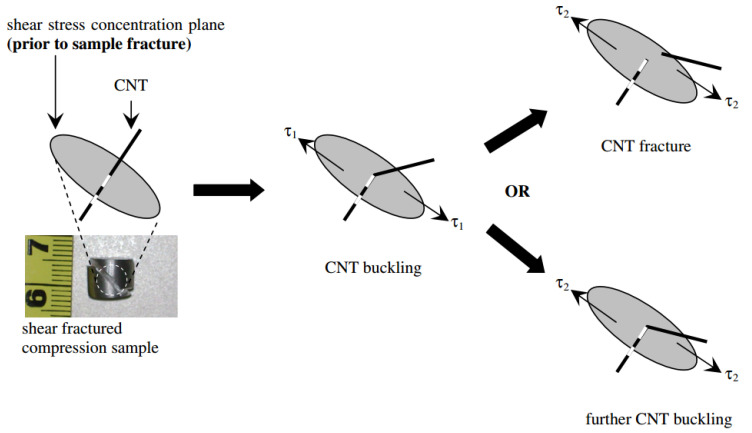
Schematic illustration of compressive shear buckling of CNTs in ZK60A/CNTs nanocomposite (τ_1_ and τ_2_ are planar shear stresses where τ_1_ < τ_2_) [100].

Recently, Sun and his co-workers [122] fabricated Mg-MWCNTs composite employing the CVD method at 480 °C utilizing acetylene gas and Co/Mg catalyst. The matrix and reinforcement powders in 1.8, 2.4, and 3 wt.% of MWCNTs loadings were BM processed at 400 rpm for 2 h under an argon-controlled atmosphere, and then sintering at 580 °C for 2 h and finally hot extrusion. The BM of the ingredient’s powders caused the CNTs to be embedded in the Mg matrix. The tensile strength of Mg was increased by raising the MWCNTs loading up to 2.4 wt.%. For the 2.4 wt.% MWCNTs loading, the tensile strength of the composite was reached to 285 MPa and was improved by about 45%, compared to the unreinforced pure Mg matrix with a tensile strength of 220 MPa. By increasing the MWCNTs content to 3 wt.%, the tensile strength declined because of the agglomeration of the MWCNTs. The HCP crystal structure of Mg is the reason for its normally inferior tensile formability. MWCNTs can activate prismatic and cross-slip dislocations in the matrix during extrusion, thus enhancing the ductility of Mg-based composites. Gupta and co-workers applied the DMD and extrusion processes for fabricating the Mg-MWCNTs nanocomposites [88,93]. They showed that the YS, UTS, and tensile elongation of Mg could be enhanced by the incorporation of 0.3 and 1.3 wt.% of MWCNTs. When increasing the MWCNTs content to 1.6 and 2 wt.%, the yield stress, tensile strength, and ductility were remarkably reduced because of the clustering of the reinforcement. The enhancement in tensile ductility of nanocomposites with MWCNTs content lower than 1.3 wt.% with a diameter of 20 nm and length of lower than 100 μm was because of the high activity of the basal slip system and the initiation of prismatic slip [93]. As shown in Figure 7, the anisotropy of tensile yield strength (TYS) or CYS could be due to the compressive shear buckling of the CNTs reinforcement in the matrix [100]. The CNTs are prone to buckling, after a possible fracture within the matrix following a compressive deformation, dissimilar to tensile deformation.

In the Ref. [100] the evaluations of compressive strength showed that for the 1 vol.% CNTs with 40–70 nm outer diameter, up to 100 aspect ratio reinforced ZK60A, the 0.2% CYS was decreased by about 14%, but the UCS was increased by about 5%, compared with the unreinforced ZK60A matrix. The compressive strength detected at any given strain was lower for 1 vol.% CNTs reinforced ZK60A compared to the ZK60A matrix. As can be seen in Figure 7, the inferior compressive strength in ZK60A/CNTs composite with 1.0 vol.% CNTs loading, compared with the unreinforced ZK60A could be due to the negative effects resulting from (a) remarkably decreased precipitation of intermetallic phase(s) in the matrix of the nanocomposite compared with the unreinforced matrix and (b) compressive shear buckling of CNTs in ZK60A/1.0 vol.% CNTs. Concerning the aforementioned reason (b), the CNT (with a high aspect ratio of about 100) is prone to buckling, followed by a fracture within the ZK60A matrix during compressive deformation [123,124]. CNTs buckling in the matrix happen more effortlessly by raising the CNTs aspect ratio. This induces a remarkably lower limit on the factors pertaining to reinforcement [100,125].

Li et al. [66] presented an efficient process for fabricating Mg/CNTs composites where the CNT possessed a diameter of around 40–60 nm and the length of lower than 2 μm. The dispersion of CNTs in the matrix was affected by the rate of solidification. For the lower rates of solidification, CNTs were pushed ahead of the solidification front and agglomerated along the boundaries of grains. Adequate bonding in interfaces of CNTs and the matrix was attained at a high solidification rate, and the UTS and YS of the composite were remarkably enhanced compared to the matrix. In other words, the superior mechanical properties of the composite were achieved at a higher rate of solidification. Park et al. [31] evaluated the mechanical properties of AZ91 Mg-alloy based composite reinforced with Si-coated MWCNTs. In this context, MWCNTs possessed an inner diameter of around 5–10 nm and the length of around 0.5–10 μm. The outcomes showed the formation of a uniform microstructure, and the performance of the composite was enhanced because of the method applied for the Si coating of the CNTs. The Si coating of MWCNTs resulted in enhancement of dispersion, wettability, and bonding strength of the CNTs in the matrix, resulting in enhancing the tensile strength of the AZ91-MWCNTs composites. Nai et al. [104] revealed that the Ni coating of CNTs (typical diameters of 10–20 nm) could result in the formation of Mg2Ni intermetallic phase at the interface of the CNTs and Mg matrix resulting in improving bonding between the reinforcement and matrix. The grain sizes were refined, and the distribution of Ni coated CNTs particles in the Mg matrix was enhanced. The aforementioned improvements resulted in simultaneously enhancing microhardness, UTS, and YS of the composite by 41%, 39%, and 64%, respectively. Despite the enhancement in mechanical characteristics of Mg/CNTs composites, the corrosion resistance of these composites has not yet been discovered in detail. In the next section, the effect of the CNTs reinforcement on the corrosion behavior of the Mg-based composites will be discussed.

**Table 1:** Mechanical properties of Mg-CNT nanocomposites.

Sample(s)	Fabrication Method(s)	Young’s Modulus (GPa)	Tensile Properties			Compressive Properties			Hardness (HV)	Ref.
			0.2%TYS (MPa)	UTS (MPa)	Elongation (%)	CYS (MPa)	UCS (MPa)	Failure Strain (%)		
Pure Mg	BM + HPS		136	163	72.1	-	-	-	-	[71]
Mg-(Ni-CNTs)	BM + HPS	0.45	454	504	10.5	505	-	-	-	[71]
Mg-1Al	SPM + VS + HTE	E_T_: 12.8 ± 0 E_C_: 5.0 ± 0.3	155 ± 3	202 ± 3	6.9 ± 0.5	100 ± 2	377 ± 8	18 ± 0.5	50 ± 4	[28]
Mg-1Al-0.60GNPs	SPM + VS + HTE	ET: 17.2 ± 0.1 EC: 7.6 ± 0.5	204 ± 9	265 ± 8	4.0 ± 0.6	230 ± 5	407 ± 3	13 ± 0.3	63 ± 2	[28]
Mg-1Al-0.60CNTs	SPM + VS + HTE	ET: 15.7 ± 0.3 EC: 6.7 ± 0.4	210 ± 5	287 ± 4	10 ± 0.3	237 ± 4	425 ± 5	12.6 ± 0.2	61 ± 5	[28]
Mg-1Al-0.60 (1:5) (CNT+GNPs)	SPM + VS + HTE	ET: 15.0 ± 0.2 EC: 6.7 ± 0.2	185 ± 4	234 ± 3	16.4 ± 0.5	167 ± 6	397 ± 3	15 ± 0.4	56 ± 3	[28]
Mg-6Zn	As-cast	-	70 ± 3.3	129 ± 2.4	8.1 ± 2.1	-	-	-	55 ± 5.8	[103]
Mg-6Zn	FSP	-	134 ± 4.8	281 ± 4.3	18.9 ± 1.1	-	-	-	69 ± 3.8	[103]
Mg-6Zn-1.0CNTs	MBM + SC + FSP	-	171 ± 2.0	330 ± 5.5	15.2 ± 1.4	-	-	-	83 ± 7.2	[103]
AZ31-0.1MWCNTs	SC + Aged	-	-	-	-	-	-	-	47	[35]
AZ31-0.5MWCNTs	SC + Aged	-	-	-	-	-	-	-	50	[35]
AZ31-1MWCNTs	SC + Aged	-	-	-	-	-	-	-	52	[35]
Mg-9Al	SPM + HTE	-	235 ± 3	301 ±5	6 ± 2	-	-	-	80.6 ± 2.8	[53]
Mg-9Al-0.2MWCNTs	SPM + HTE	-	242 ± 3	346 ± 4	14 ± 1	-	-	-	91.8 ± 1.9	[53]
Mg-9Al 0.4MWCNTs	SPM + HTE	-	248 ± 5	355 ± 7	15 ± 3	-	-	-	94.2 ± 2.7	[53]
Mg-9Al-0.6MWCNTs	SPM + HTE	-	230 ± 2	329 ± 1	13 ± 1	-	-	-	89.6 ± 4.8	[53]
AZ91	SPM + HTE	-	168 ± 5.0	215 ± 6.0	7.0 ± 0.2	-	-	-	72.4 ± 2.0	[69]
AZ91-1CNT	SPM + HTE	-	173 ± 4.0	228 ± 5.0	8.6 ± 0.1	-	-	-	79.2 ± 2.0	[69]
AZ91-2CNT	SPM + HTE	-	197 ± 4.5	263 ± 5.5	8.7 ± 0.2	-	-	-	87.1 ± 1.5	[69]
AZ91-3CNT	SPM + HTE	-	250 ± 3.8	301 ± 4.5	9.4 ± 0.1	-	-	-	94.1 ± 2.0	[69]
AZ91-4CNT	SPM + HTE	-	187 ± 3.5	248 ± 3.9	8.5 ± 0.1	-	-	-	84.3 ± 1.6	[69]
AZ91-5CNT	SPM + HTE	-	154 ± 4.4	228 ± 5.6	7.8 ± 0.2	-	-	-	80.2 ± 1.7	[69]
AZ91-1(MgO-CNT)	SPM + HTE	-	190 ± 3.6	260 ± 4.2	7.6 ± 0.1	-	-	-	80.2 ± 1.5	[69]
AZ91-2(MgO-CNT)	SPM + HTE	-	210 ± 5.0	294 ± 6.0	8.2 ± 0.2	-	-	-	89.5 ± 1.0	[69]
AZ91-3(MgO-CNT)	SPM + HTE	-	284 ± 4.6	331 ± 5.0	8.6 ± 0.1	-	-	-	96.4 ± 1.2	[69]
AZ91-4(MgO-CNT)	SPM + HTE	-	206 ± 3.7	272 ± 4.8	8.0 ± 0.1	-	-	-	86.5 ± 1.2	[69]
AZ91-5(MgO-CNT)	SPM + HTE	-	175 ± 5.5	255 ± 5.0	7.4 ± 0.2	-	-	-	83.6 ± 1.5	[69]
AZ91D-2 CNT	SC	-	-	290.4	-	-	-	-	80.23	[99]
AZ91D- 3CNT	SC	-	-	300.456	-	-	-	-	85.91	[99]
AZ91D-4CNT	SC	-	-	295.68	-	-	-	-	92.3	[99]
Mg- 0.08CNTs	PM + SPS + HTE	-	185	238	16.1	-	-	-	-	[57]
AZ31-1CNTs	BM + Extrusion + Welding	-	186 ± 5.6	272 ± 7.2	6 ± 1.5	-	-	-	67 ± 3.6	[36]
AZ91D	SC	-	202	264	-	-	-	-	71	[98]
AZ91D-2CNT	SC	-	216.14	289.23	-	-	-	-	79.49	[98]
AZ91D-3CNT	SC	-	228.26	296.47	-	-	-	-	83.78	[98]
AZ91D-4CNT	SC	-	222.20	293.35	-	-	-	-	90.88	[98]
Mg-3Al-1Zn	SPM	44.1	149	248	15.24	-	-	-	46.36 ± 3.6	[126]
Mg-3Al-1Zn -0.5CNTs	SPM	50.2	163	267	15.91	-	-	-	57.00 ± 4.0	[126]
Mg-3Al-1Zn -1.0CNTs	SPM	55.5	184	296	22.59	-	-	-	61.38 ± 2.2	[126]
Mg-3Al-1Zn -1.5CNTs	SPM	52.4	176	260	20.39	-	-	-	61.88 ± 3.3	[126]
ZK60A	DMD	-	163 ± 3	268 ± 3	6.6 ± 0.6	128 ± 11	522 ± 11	19.6 ± 0.9	138 ± 7	[100]
ZK60A-1.0CNT	DMD	-	180 ± 6	295 ± 8	15.0 ± 0.7	110 ± 7	547 ± 3	33.2 ± 6.2	114 ± 6	[100]
Mg–6Zn-0.5 CNT	BM + Ultrasonic treatment + SC	-	92	192	-	-	-	-	-	[29]
AZ91-0.1 MWCNTs	SC	-	-	-	-	161 ± 4.5	412	24.4	-	[34]
AZ31	PM + Extrusion	-	195 ± 5.0	285 ± 2.9	14.5 ± 1.5	160 ± 6.0	363 ± 3.5	16.3 ± 1.5	58 ± 3.0	[55]
AZ31-0.3GNP	PM + Extrusion	-	173 ± 6.2	275 ± 5.7	21.7 ± 2.8	161 ± 4.5	397 ± 5.3	16.0 ± 1.8	71 ± 2.1	[55]
AZ31-0.3 CNT	PM + Extrusion	-	210 ± 2.8	310 ± 5.4	13.3 ± 3.0	242 ± 5.5	457 ± 6.0	14.0 ± 1.3	78 ± 2.8	[55]
Mg-6Al-0.5CNT	MBM + CP + HTE	-	-	-	-	-	~160	-	~40	[92]
Mg-6Al-1CNT	MBM + CP + HTE	-	-	-	-	-	~140	-	~36	[92]
Mg-6Al-2CNT	MBM + CP + HTE	-	-	-	-	-	~105	-	~34	[92]
Mg-6Al-4CNT	MBM + CP + HTE	-	-	-	-	-	~75	-	~28	[92]
Mg-0.05CNT with 20% overall porosity	PM	-	71.5 ± 19.5	-	-	-	-	-	-	[72]
Mg-0.05CNT with 30% overall porosity	PM	-	48 ± 18	-	-	-	-	-	-	[72]
Mg-0.05CNT with 40% overall porosity	PM	-	20 ± 8	-	-	-	-	-	-	[72]
Mg-1CNT with 20% overall porosity	PM	-	87.5 ± 25.5	-	-	-	-	-	-	[72]
Mg-1CNT with 20% overall porosity	PM	-	51.5 ± 19.5	-	-	-	-	-	-	[72]
Mg-1CNT with 20% overall porosity	PM	-	24.5 ± 10.5	-	-	-	-	-	-	[72]
AZ81	DMD + HTE	-	225	336	7.9	157 ± 17	487 ± 14	17.0 ± 0.1	119± 2	[61]
AZ81-1.5CNTs	DMD + HTE	-	280	392	12.9	129 ± 19	488 ± 13	16.0 ± 1.8	114 ± 8	[61]
Mg-0.05CNTs	EPD + HPS + HR	-	115 ± 4.0	153 ± 4.5	4.6 ± 0.9	-	-	-	-	[73]
Mg-0.10 CNTs	EPD + HPS + HR	-	143 ± 7.8	172 ± 2.6	5.5 ± 0.9	-	-	-	-	[73]
Mg (98.5% Purity)	MBM + CP + HTE	-	127 ± 5	205 ± 4 9	9 ± 2	-	-	-	45 ± 0	[30]
Mg-0.06 CNTs	MBM + CP + HTE	-	133 ± 2	203 ± 1	12 ± 1	-	-	-	44 ± 0	[30]
Mg-0.18 CNTs	MBM + CP + HTE	-	138 ± 4	206 ± 7	11 ± 1	-	-	-	44 ± 1	[30]
Mg-0.30 CNTs	MBM + CP + HTE	-	146 ± 5	210 ± 6	8 ± 1	-	-	-	44 ± 0	[30]
Mg-2 wt.% CNTs	BM + HPS	38.6 ± 0.7	89	140	3	-	-	-	-	[75]
Mg (98.5% Purity)	BM + MS + HTE	-	126 ± 1	171 ± 2	7.9 ± 0.3	-	-	-	39 ± 3	[104]
Mg-0.3CNTs	BM + MS + HTE	-	119 ± 4	163 ± 7	5.7 ± 0.2	-	-	-	36 ± 1	[104]
Mg-0.3 (Ni-CNTs)	BM + MS + HTE	-	206 ± 2	237 ± 1	6.4 ± 0.3	-	-	-	55 ± 3	[104]
AZ91	SI	5 ± 2	80 ± 5	205 ± 5	-	-	-	-	80	[31]
AZ91-5MWCNTs	SI	1.0 ± 2	210 ± 10	243 ± 10	-	-	-	-	150	[31]
AZ91-5 (Si-MWCNTs)	SI	1.3 ± 2	253 ± 10	296 ± 10	-	-	-	-	160	[31]
Mg	PM + HTE	-	-	-	-	106 ± 11	239 ± 15	19.8 ± 1.7	40 ± 2	[70]
Mg-0.5Al-0.18CNT	PM + HTE	-	-	-	-	120 ± 09	357 ± 13	11.0 ± 1.3	50 ± 4	[70]
Mg-1Al-0.18CNT	PM + HTE	-	-	-	-	132 ± 04	421 ± 15	12.5 ± 1.0	58 ± 3	[70]
Mg-1.50Al-0.18CNT	PM + HTE	-	-	-	-	144 ± 07	421 ± 11	11.3 ± 1.7	60 ± 4	[70]

CNTs: Carbon nanotubes, MWCNTs: Multi-walled carbon nanotubes, GNPs: Graphene nanoplatelets, ET: Elastic modulus in tensile, EC: Elastic modulus in compressive, TYS: Tensile yield strength, UTS: Ultimate tensile strength, CYS: Compressive yield strength, UCS: Ultimate compressive strength, HPS: Hot pressing sintering, BM: Ball milling, SPM: Semi-powder metallurgy, HTE: Hot extrusion, VS: Vacuum sintering, DMD: Disintegrated melt deposition, PM: Powder metallurgy, MBM: Mechanical ball milling, SC: Stir Casting, FSP: Friction stir processing, SPS: Spark plasma sintering, CP: Cold pressing, EPD: Electrophoretic deposition, HPS: Hot press sintering, HR: Hot rolling, MS: Microwave sintering, SI: Squeeze casting infiltration.

## 6. Corrosion Properties

Magnesium presents considerable potential as a lightweight material for several purposes in the automobile, aviation, and medical fields. However, Mg’s usage has been hindered because of low corrosion resistance that leads to a reduction in their functionality throughout servicing life in a corrosive solution. Although it has been found that, generally, the mechanical characteristics of CNT-reinforced MMCs are higher than that of their monolithic matrix, the corrosion properties of these composites have not yet been discovered in detail [88,91,93]. Because of a low standard electrode potential (−2.36 V), and inferior protecting property of the formed magnesium oxide or magnesium hydroxide layers in different environments, Mg and its alloys display a comparatively high corrosion rate. Due to having a low standard potential, the coupling Mg with almost all other materials which are interesting technologically, makes the resulting composite very prone to galvanic corrosion [126]. Conventional ceramic particulates or carbon fiber-reinforced MMCs have poor corrosion resistance when exposed to corrosive environments like the solutions that contain chloride ions. In these composites, the interfaces of reinforcement and matrix are especially prone to be attacked by chloride ions, resulting in pitting and crevice corrosion [127]. The existence of SiC [128], Al_2_O_3_ [129], Cu [130], and Mo [130] reinforcements can also increase the corrosion rate of Mg-based composites. For the MWCNT-reinforced MMNCs, depending on the process employed for fabricating the composite and nature of the matrix material, the corrosion rate may increase or decrease. For the Mg-based nanocomposites, the incorporation of CNTs reinforcement increases the corrosion rate [63,131,132]. The corrosion rate increases because of a big difference between the standard electrode potentials of the Mg-based matrix and CNTs, resulting in the formation of galvanic couples between them. In Mg alloys, intermetallic phases like Al–Mn–Fe, β-Mg_17_Al_12_, and Al–Mn act as active cathodes to cause micro-galvanic corrosion at the anodic Mg-based matrix [88,133,134,135]. Incorporating elemental carbon, with a very higher standard electrode potential than Mg [136], can also cause the galvanic corrosion in the Mg/CNTs composite.

It is reported [64] that polyacrylonitrile-based carbon fibers declined the corrosion resistance of Mg because of the forming of galvanic cells between carbon fibers and Mg. Nevertheless, CNTs have relatively different electrical properties from the other allotropes of carbon. Hence, the effect of CNTs on the corrosion of Mg could be different from that of other allotropes of carbon [11]. Endo et al. [76] argued that the MWCNTs could keep the oxide layer detached from the Mg alloy, and it could slow down the further forming of the oxide layer. The researchers revealed that the corrosion rate of AZ91D-CNTs composite was decreased compared to that of original AZ91D Mg alloy in saltwater because of the formation of stable oxide films in the grain boundaries of Mg. They showed that CNTs have a water protection property and reinforce the surface protective layers. Through both phenomena, the corrosion resistance should be enhanced, and no specific effort was made to discover the role of galvanic corrosion between CNTs and the Mg-based matrix. On the other hand, it was uncertain whether the CNTs could enhance the corrosion resistance because of the forming of a stable oxide film. The corrosion behavior of MMCs is mainly dependent on the possible formation of a passive layer on the surface of the composite, which can protect it [11]. Research showed no passivation behavior on Mg/CNTs composite for preventing further corrosion [137]. The observation of the microstructure showed that the surface of the composite was locally damaged, and lots of CNTs existed at the primary particle boundary, and severe corrosion happened in the vicinity of CNTs due to the galvanic corrosion [138,139]. Fukuda et al. [64] investigated the corrosion behavior of the SPS-fabricated AZ31B-MWCNT composites exposed to NaCl solutions applying immersion and polarization examinations. The formation of the galvanic cell between the CNTs and a Mg-based matrix in NaCl solution because of the significant potential difference occurred, and a large amount of corrosion products were piled up in the vicinity of the CNTs resulting in decreasing the corrosion resistance of the AZ31B Mg alloy. The immersion examination showed that the pH value of the test solution quickly increased after immersion of AZ31B-MWCNT composite and reached a higher value than that of the unreinforced AZ31B alloy. The reactions of monolithic Mg in aqueous solution are given by [140,141,142]:

Mg →Mg^2+^ + 2e^−^    (anodic reaction) (2)

2H_2_O + 2e^−^ → H_2_ + 2OH^−^ (cathodic reaction) (3)

(4) $$Mg^{2+}+2OH^{-}\to Mg{\left(OH\right)}_{2}\;(\mathrm{corrosion}\;\mathrm{product})$$

Based on the aforementioned reactions, the dissolution of Mg causes a rise in the solution pH. Moreover, the layer of Mg(OH)_2_ on the surface is not so restrictive and could not provide adequate protection for preventing the corrosion in the aqueous environment. The Cl^−^ ions could change the Mg(OH)_2_ into the soluble MgCl_2_ salt. This reaction makes the surface of the composite more active, and decreases the protected area, resulting in increasing the dissolution of Mg, based on the reactions:

(5) $$Mg+2Cl^{-}\to MgCl_{2}$$

(6) $$Mg{\left(OH\right)}_{2}+2Cl^{-}\to MgCl_{2}+2OH^{-}$$

Aung et al. [63] revealed that the corrosion rate of Mg/CNTs composite increased compared with that of monolithic Mg, applying hydrogen evolution measurement, mass loss during immersion tests, and polarization curves in NaCl solution. They claimed that the reason was the galvanic corrosion between the CNTs and the Mg matrix. In other studies, Li et al. [132], Turhan et al. [131] and Aung et al. [63] showed that the corrosion resistance is significantly decreased by incorporating the CNTs to Mg alloy because of the formation of galvanic couples. Table 2 shows the summarized information about the corrosion behavior of Mg/CNTs composites.

For controlling the galvanic corrosion, it is necessary to decrease the potential difference at the interface of CNTs and the Mg matrix. Funatsu et al. [143] presented an efficient method for improving the corrosion resistance of Mg/CNTs composite. They produced AZ61B-CNTs composite applying the PM process and reported that the potential difference between CNTs and the α-Mg matrix was decreased by the concentration of Al atoms around CNTs using a heat treatment at 823 K for 10 h. The results of immersion examination in NaCl solution revealed that the corrosion rate of AZ61B-CNTs composite after heat treatment was remarkably decreased to less than about 30% of the untreated composite. Aydin et al. [89] investigated plasma electrolytic oxidation (PEO)-coated Mg/MWCNTs composites applying potentiodynamic corrosion tests. They revealed that the corrosion rate of the coated specimen was about 1.6 times less than that of the uncoated composite.

**Table 2:** Corrosion properties of carbon nanotube-reinforced magnesium nanocomposites.

Samples	Reinforcement	Processing Route	Reinforcement Particle Size	Corrosion Medium	i_corr_ (μA·cm^−2^)	E_corr_ (V vs. SCE)	Corrosion Rate (mm/year)			Rp (Ω·cm^2^)	Ref.
							Non Polarized		Polarized		
							Immersion Time (h)	HE or WL	PDP		
AZ31 (after 1, 24 and 336 h immersion time)	-	DMD	-	SBF	94.70	–1.545	1	2.176 mm/year	-	-	[144]
			-		44.22	–1.441	24	1.016 mm/year	-	-	
			-		213.45	–1.402	336	4.906 mm/year	-	-	
AZ31 (after 1, 24 and 336 h immersion time)	CNT (1wt.%)		-		87. 62	–1.502	1	2.013 mm/year	-	-	
			-		17.42	–1.408	24	0.400 mm/year	-	-	
			-		9.33	–1.373	336	0.214 mm/year	-	-	
Mg–6Al	CNT (4 wt.%)	MBM + CP + HTE	average diameter: 9.5 nm average length: 1.5 μm	3.5% NaCl	5	–1.55	-	-	-	-	[92]
AZ91	MWCNTs (1 wt.%)	MS	-	3.5% NaCl	-	–	-	5–9 gm^−2^day^−1^	-	-	[132]
	MWCNTs (5 wt.%)		-		-	–	-	19–24 gm^−2^day^−1^	-	-	[132]
Mg	MWCNTs (0.1 wt.%)-Dispersed during melt stirring process (0 h)	MS	-	3.5% NaCl	98	–1.618	-		4.5 mm/year	368	[131]
	MWCNTs (0.1 wt.%)- Dispersed during melt stirring process (6 h)		-		280	–1.520	-		12.8 mm/year	332	
AZ91	GNPs (0.5 wt.%)	SPM	diameter: 5 and 8 nm surface area: 750 m^2^/g	3.5 wt.% NaCl	326.902 μA	–1.449	-	-	4.13 mm/year	-	[145]
	MWCNT (0.5 wt.%)		diameter: 8 nm surface area: 250 m^2^/g		388.431 μA	–1.491	-	-	4.92 mm/year	-	
	C60 (0.5 wt.%)		average thickness: 1–2 nm		212.137 μA	–1.506	-	-	2.68 mm/year	-	
Mg	CNT (0.3wt.%)	DMD	average diameter: 20 nm and length: less than 100 μm	3.5 wt.% NaCl	56	–1.57	-	-	-	-	[63]
	CNT (1.3wt.%)				572	–1.50	-	-	-	-	
Mg	MWCNTs (0.5 wt.%)	SPM	-	3.5 wt.% NaCl	579.4 μA	–1.544	-	-	24.62 mm/year	-	[89]
Mg	MWCNTs (0.5 wt.%)	SPM + PEO	-		130.4 μA	–1.401	-	-	14.78 mm/year	-	
Mg-0.5 MWCNT	GNP	SPM + PEO (coating with graphene addition)	-		101.0 μA	–1.424	-	-	14.46 mm/year	-	

CNTs: Carbon nanotubes, MWCNTs: Multi-walled carbon nanotubes, GNPs: Graphene nanoplatelets, I_corr_: Corrosion current density, E_corr_: Corrosion potentials, HE: Hydrogen evolution, WL: Weight loss, PDP: Potentio-dynamic polarization, Rp: Polarization resistance, DMD: Disintegrated melt deposition, PM: Powder metallurgy, SPM: Semi powder metallurgy, HTE: Hot extrusion, MBM: Mechanical ball milling, CP: Cold pressing, MS: Melt stirring, SLM: Selective laser melting, PEO: Plasma electrolytic oxidation.

## 7. Wear and Friction Properties

Due to the increasing demand for lightweight materials in various fields, including automotive and biomedical applications, Mg-based composites have developed quickly. Regardless of their wonderful features according to outstanding machinability, Mg’s low hardness and inferior tribological characteristics have remarkably restricted the usage of this material for non-tribological fields. Insufficient hardness and wear resistance of Mg is elevated via various approaches, including the fabrication of Mg-based composites as a cost-effective manufacturing technique. Although incorporating the ceramic reinforcements can enhance the wear resistance of MMCs, they may raise the coefficient of friction (COF) [11]. CNTs with superior mechanical properties and self-lubricating behavior are an appropriate reinforcement to fabricate the MMNCs with low COF and high wear resistance [11,145,146,147]. In the past decade, researchers’ attention has increasingly been attracted to CNTs as an ideal reinforcement for fabricating the Mg-based composites, and research has been mostly concentrated on studying the CNTs’ effects on the improvement of the mechanical and corrosion properties of structural parts. Moreover, the wear behavior of Mg-CNTs nanocomposites has been evaluated in some studies.

A remarkable enhancement was seen in wear resistance of MWCNT-reinforced AZ31 Mg–alloy composite fabricated by FSP, comparing with that of unreinforced AZ31 alloy. Homogenous distribution of MWCNTs reinforcement in the matrix, grain refinement, and high interfacial bonding at the MWCNTs and AZ31 matrix interface was claimed as the reasons for the achieved enhancement of the mechanical properties [102]. Selvamani et al. [99] investigated the wear behavior of AZ91D-CNTs nanocomposites fabricated by the stir casting process. Three different loadings of 2, 3, and 4 wt% of CNTs reinforcement were chosen for preparing the composites, and 4 wt% CNT-reinforced composite showed higher hardness value while the one with 3 wt% of CNTs exhibited superior wear resistance comparing with the AZ91D matrix, and it showed a fracture with a ductile mode because of the improved tensile strength compared to the other composites.

**Figure 8:**
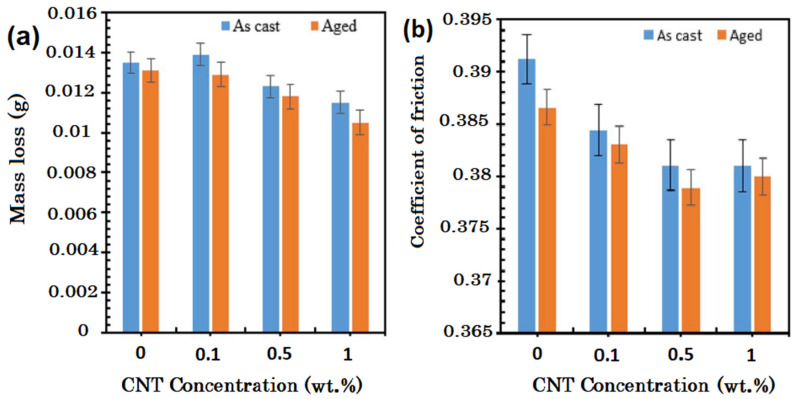
Plot of the changes of (**a**) wear mass loss, and (**b**) coefficient of friction against the concentration of CNTs in AZ31-CNTs composite under dry sliding conditions [35].

Abbas et al. [35] studied the wear behavior of AZ31-MWCNTs composites fabricated by stir casting, followed by age hardening for 10 h. The results of the wear test, including the plots of mass loss and coefficient of friction against the ascending wt% of CNTs, are depicted in Figure 8 [35]. Their outcomes revealed that the wear weight loss reduced with increasing CNT concentration. This can be attributed to two main reasons: (1) improving the strength and hardness of the composite resulted by the addition of CNTs, and (2) decrease in the friction coefficient due to the self-lubricating effects CNTs, that can decrease the wear mass loss. During wear, the CNTs are pulled out, and a layer of carbon is shaped between the counterface and composite, which can act as a solid lubricant, leading to a decrease in the coefficient of friction and rate of wear. Moreover, the outcomes showed that the aged composites had a comparatively inferior friction coefficient compared with the as-cast composites [35].

Mindivan et al. [92] showed that a lubricating effect could be provided by the coarse debris and/or CNTs pulled out from the Mg–6Al- CNTs composite at the interface of the steel ball and surface of the composite material. Hence, the composite with an inferior value of hardness may display a poor wear resistance at a higher loading of CNTs if there would not be an adequate bonding between the chip layers. A loose bonding reduces the direct contact between the Mg-based matrix and the steel ball because of the effortless detachment of CNTs from the worn surface.

**Figure 9:**
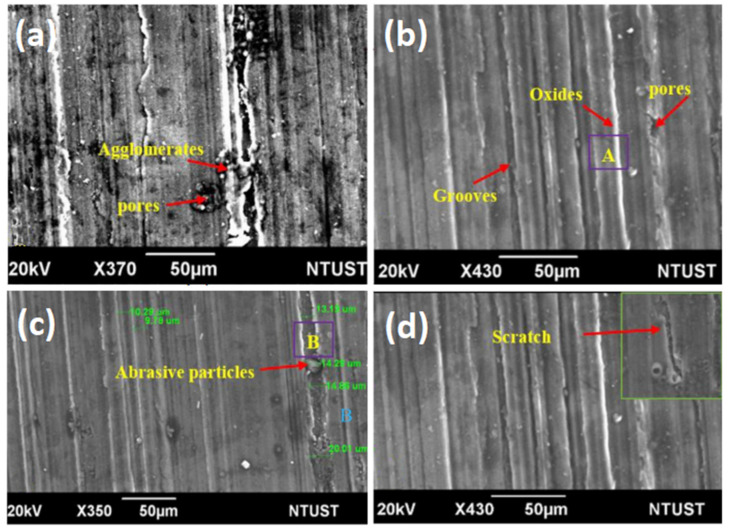
Scanning electron microscope (SEM) images of worn surfaces of AZ31-CNTs composites including (**a**) 1.0 wt.%, (**b**) 0.5 wt.%, (**c**) 0.1 wt.%, and (**d**) 0 wt.% of CNTs, under dry sliding conditions [35].

The wear mechanism of AZ31-CNTs composites containing different concentrations of CNTs was investigated by analyzing the morphology of worn surfaces, applying scanning electron microscopy (SEM) [35]. The mechanisms of abrasion, delamination, oxidation, and very slight plastic deformation was seen on the worn surfaces of the parts. Figure 9 shows the SEM micrographs of worn surfaces under dry sliding conditions after tribological testing [35]. The abrasion can be seen in Figure 9a, which is related to the composite containing 1 wt% CNTs. As can be seen, many scratches and grooves are observable that most of them are in sliding direction. Some CNTs reinforcement particles in this sample, with comparatively higher loading of CNTs, may be agglomerated and act as plowing tools for the material and result in abrasive wear. The mechanism of three-body abrasion happens when an adequately hard particle is trapped between two surfaces which one of them, or both could be abraded by the particle [102]. This wear mechanism could occur under any conditions of loading and could occur at the same time with all other wear mechanisms [148]. Mg alloys typically have a low oxidation resistance, and their oxidation can affect the wear rates. The friction on contact surfaces can generate heat, which can facilitate the oxidation. Repeating slide motions can break some fragile magnesium oxides into fragments and cause a protective layer on the surface, which can avoid direct contact of sliding surfaces. In Figure 9b, which is related to the composite containing 0.5 wt% of CNTs, the oxidation wear could be observed. The oxidation thickness strongly influences the wear mechanism. A thick layer of magnesium oxide protects sliding surfaces and provides a mild wear condition with low wear rates [149]. The delamination wear is dependent on fatigue. Dynamic forces on the sliding surfaces create some cracks that propagate after nucleation. The formed cracks can shear the surfaces, resulting in the removal of sheet-like laminates, leaving channels or caves on the worn surface, as can be seen in Figure 9c,d, which are related to the composite containing 0.1% CNTs and unreinforced AZ31 Mg alloy, respectively. Many deformations of sheet-like delaminated wear debris leaving shallow craters can be observed in Figure 9c. Increasing the temperature on contact surfaces is the most significant parameter for determining the wear mechanism. Rising temperature accelerates the softening and sticking of asperities to the parts. The results revealed no adhesion to the counterpart, and abrasive asperities were accumulated on the contact surface after repeated sliding, resulting in nucleation beneath the surfaces and detachment of laminates. Plastic deformation as a severe type of wear mechanism happens when a large surface is worn without the formation of cracks, and it causes extensive surface damage. In Figure 9d the mechanism of plastic wear can be seen. This mechanism is a transition from delamination to plastic deformation at higher loads and rates [150]. The transition from the mild to the severe wear regime is accomplished by increasing the surface roughness [151]. The temperature between the steel counter face and the test specimen reaches high levels to assist plastic wear behavior. Consequently, the plastic deformation of the surface is facilitated in the sliding direction and with sideways motion [152]. The large irregular lumps which are detached from the part are severely deformed and extruded along the direction of sliding [35]. The incorporation of CNT reinforcement in MMCs plays a crucial role in the improvement of wear behavior and a reduction in the COF of the composite. The influence of CNTs on the wear behavior of Mg/CNTs composites has been investigated, but additional studies are needed for optimizing the relationship between the CNTs loading and the relevant wear behavior.

## 8. Summary and Future Road Maps

The present review article aimed to provide an overview of the current progress, possibilities, challenges, and upcoming exploration of the CNT-reinforced Mg-based composites. CNTs have various outstanding characteristics, such as high aspect ratio, exceptionally high strength and Young’s modulus, and very high thermal and electrical conductivity. The aforementioned properties have attracted great attention for using CNTs as a reinforcement of MMCs for enhancing the properties of the composites, including higher strength, lower weight, and self-lubricating behavior. The advantageous properties could be attained only if the reinforcements are distributed homogeneously and not clustered in the matrix. According to the literature available [153,154,155,156,157,158,159,160,161,162,163,164,165,166,167,168,169,170,171,172,173,174,175,176,177,178,179,180,181,182,183,184,185,186,187], limited research has been performed on CNT-reinforced MMCs due to difficulties in fabricating and dispersion of CNTs in the matrix. Most studies investigated the fabrication methods such as the casting process and powder metallurgy for developing Mg-based alloy [188,189,190,191,192,193,194,195,196,197,198,199,200,201,202,203,204,205,206,207,208,209,210,211,212,213,214], some of them focused on mechanical properties, and some studied the self-lubricating behavior Mg/CNTs-based composites. Based on the studies, the mechanical characteristics of the composites were significantly improved by the addition of CNTs. The ball mill process seems to be an efficient process to achieve the improved dispersion of CNTs in the Mg-based matrix. The interfaces of CNTs and the matrix can affect the efficiency of load transfer from the Mg-based matrix to the CNTs’ reinforcement during mechanical deformation. Appropriate interfacial bonding is crucial for ensuring an efficient load transfer between the matrix and CNT reinforcements. Normally, CNTs could not be properly wetted by the Mg-based matrix during the fabrication process of the composite. Hence, the coating of CNTs with Cu, Ni, or Cr could enhance the wettability of the CNTs. The micro-galvanic effect is the main corrosion mechanism of Mg/CNT composites in sodium chloride solution. The corrosion resistance of the Mg/CNT composites was considerably decreased with a rise in the concentration of CNTs. The primary outcomes suggested that the CNTs acted as efficient cathodes to promote the corrosion rate. Further investigations to find the role of CNTs in the corrosion resistance of Mg and its alloys would be an upcoming effort of the present study. The resulting material characteristics of CNT-reinforced Mg-based composites are related to the uniform distribution of CNTs, interfacial bonding between CNTs and the matrix, CNTs’ geometry and concentration, and their alignment with the matrix. At the same time, the properties are a function of the parameters of the fabrication process employed for preparing the composites. Nevertheless, many studies remain to be done to optimize the fabrication processes, microstructure properties, and evaluation of composite performance before using an Mg/CNT composite in commercial applications.

## Abbreviations

Abbreviations		Abbreviations	
BM	Ball milling	I_corr_	Corrosion current density
CIP	Cold isostatic pressing	MBM	Mechanical ball milling
CNT	Carbon nanotube	MMCs	Metal matrix composite
COF	Coefficient of friction	MMNCs	Metal matrix nanocomposites
CP	Cold pressing	MS	Microwave sintering
CTE	Coefficient of thermal expansion	MWCNT	Multi-walled carbon nanotube
CVD	Chemical vapor deposition	PDP	Potentiodynamic polarization
CYS	Compressive yield strength	PEO	Plasma electrolytic oxidation
DMD	Disintegrated melt deposition	PM	Powder metallurgy
E_C_	Elastic modulus in compressive	Rp	Polarization resistance
ECAP	Equi-channel angular processing	SC	Stir Casting
E_corr_	Corrosion potentials	SHE	Standard hydrogen electrode
EPD	Electrophoretic deposition	SI	Squeeze casting infiltration
E_T_	Elastic modulus in tensile	SPM	Semi powder metallurgy
FSP	Friction stir processing	SPS	Spark plasma sintering
FSW	Friction stir welding	SWCNT	Single-walled carbon nanotube
GNP	Graphene nanoplatelet	TYS	Tensile yield strength
HCP	Hexagonal close-packed	UCS	Ultimate compressive strength
HE	Hydrogen evolution	UTS	Ultimate tensile strength
HEBM	High-energy ball milling	VS	Vacuum sintering
HIP	Hot isostatic pressing	WL	Weight loss
HPS	Hot-press sintering	YS	Yield strength
HR	Hot rolling		
HTE	Hot extrusion		
